# Laser etching of groove structures with micro-optical fiber-enhanced irradiation

**DOI:** 10.1186/1556-276X-7-318

**Published:** 2012-06-19

**Authors:** Dameng Liu, Jiachen Liu, Hui Wang, Tianmin Shao

**Affiliations:** 1 State Key Laboratory of Tribology, Tsinghua University, Beijing, 100084, China

**Keywords:** Micro-fiber, Laser, Micro-fabrication

## Abstract

A microfiber is used as a laser-focusing unit to fabricate a groove structure on TiAlSiN surfaces. After one laser pulse etching, a groove with the minimum width of 265 nm is manufactured at the area. This technique of microfabricating the groove in microscale is studied. Based on the near-field intensity enhancement at the contact area between the fiber and the surface during the laser irradiation, simulation results are also presented, which agree well with the experimental results.

## Background

In the past 10 years, with the important role of microdevices in industrial applications, fabrication technologies on the micron and nanometer scales are attracting more attentions. Recently, developed lithographic technologies like ion beam, electron beam, and near-field optics have been investigated and regarded as potential methods for microfabrication [1-4]. Due to the light diffraction limit, traditional laser optical manufacture is not available to fabricate the further microsturctures [5]. Many groups offered approaches to overcome the limit by using near-field effects through surface pattern designs. These are carried out by delivering a laser beam through a near-field tip or illuminating the tip with a pulsed laser. Huang et al. used single-pulse 248 nm KrF laser radiation to fabricate nano bump arrays [6]. Hong et al. reported that femtosecond laser (400 nm, 100 fs) irradiation went through a near-field scanning optical microscope and sub-50 nm feature size was created [7]. The near-field enhanced laser irradiation with 248 and 355 nm UV lasers was applied to pattern a silicon surface in a massively parallel fashion [8]. However, these approaches need the expensive equipment with the related complex system. Zhang et al. used a 248 nm excimer laser with a pulse duration of 23 ns and obtained 100 nm hillocks at the original particle positions [4]. These glass microballs can only be used for processing micro-dimple structures. Zhou et al. also tried a low-cost method, in which an optical fiber was used as a focusing unit to etch parallel microgrooves with 2 to 6 μm width and 0.7 to 1.4 μm depth on Si surface [9]. This resolution still needed to be improved to overcome the diffraction limit. Meanwhile, it is necessary for the micro and nanofabrication to develop the low-cost and fast speed laser processing [10]. To further reduce the resolution to the nanometer scale, methods of near-field laser irradiation with the combination of advanced processing tools such as SPM, NSOM, transparent, and metallic particles are applied for the sizes as small as 20 nm. Laser interference lithography is also capable of fabricating sub-100 nm periodic structures for large area, maskless, and noncontact nanofabrications [7]. In this work, we report a low-cost microprocessing technique, based on a microfiber-enhanced irradiation to fabricate groove structures under the diffraction limit.

## Methods

**Figure 1:**
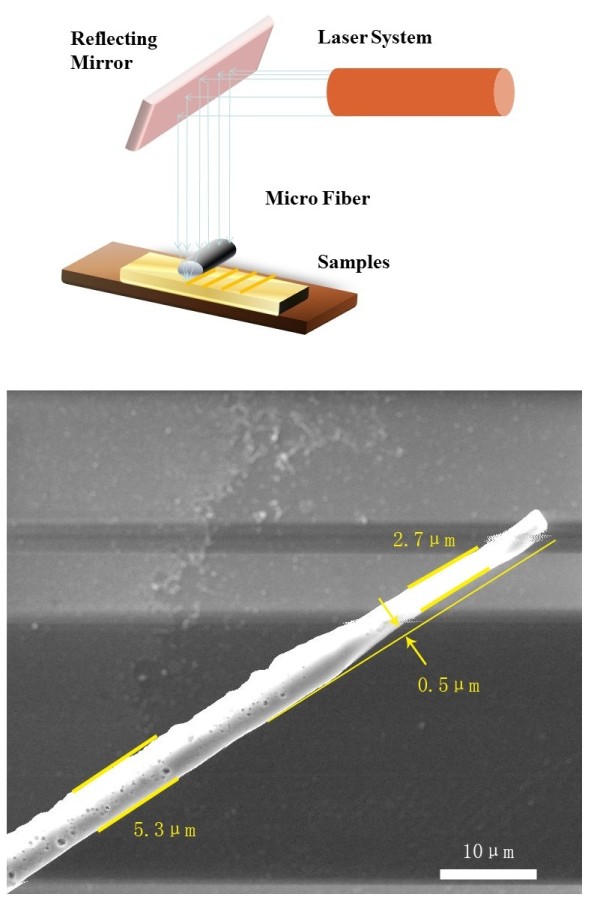
Diagram of the laser etching system based on microfiber-enhanced irradiation.

A laser etching experiment with the microfiber-enhanced irradiation is designed to fabricate a groove on TiAlSiN surfaces (Figure 1). Nd: YAG solid state laser at a wavelength of 1.06 μm, repetitive rate of 1 to 10 Hz, and laser beam diameter of 7 mm is applied in our experiment as a light source. This fiber was fabricated from a quartz optical fiber. The original fiber's diameter is 125 μm, with the refractive index of 1.588. The ultrasound process is used for the quartz fiber in order to remove its plastic covers in sulfuric acid. Then, the fiber is washed in DI water and dipped into hydrofluoric acid solution (47%) for about 50 min. Finally, a fiber in microscale is obtained and examined under a high resolution scanning electron microscope (SEM).

In the groove manufacture process, the micro-fiber is placed on the TiAlSiN surface. The fiber contacts with the surface directly, which makes the gap between the fiber and the surface at nanometer scales. Light intensity at the sample surface is adjusted at 440 mJ/cm^2^. The laser perpendicularly irradiates on TiAlSiN through the fiber and etches the surface. After one laser pulse etching, a groove on the TiAlSiN surface is created. It is observed under both SEM and atomic force microscopy (AFM).

RF module of Comsol 3.5 is used to simulate optical fields. Here, it is required to calculate the energy distribution behind the fiber. The calculations are based on finite element analysis to solve Maxwell equations with the boundary conditions. The calculation results are compared to the groove characters.

## Results and discussion

**Figure 2:**
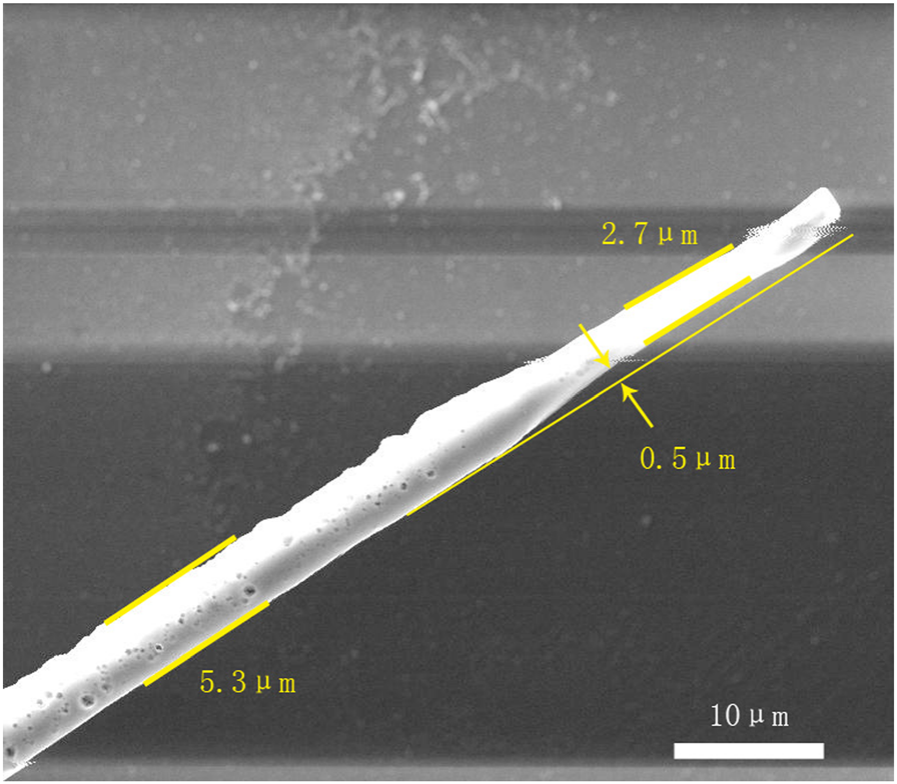
SEM image of the micro fiber.

**Figure 3:**
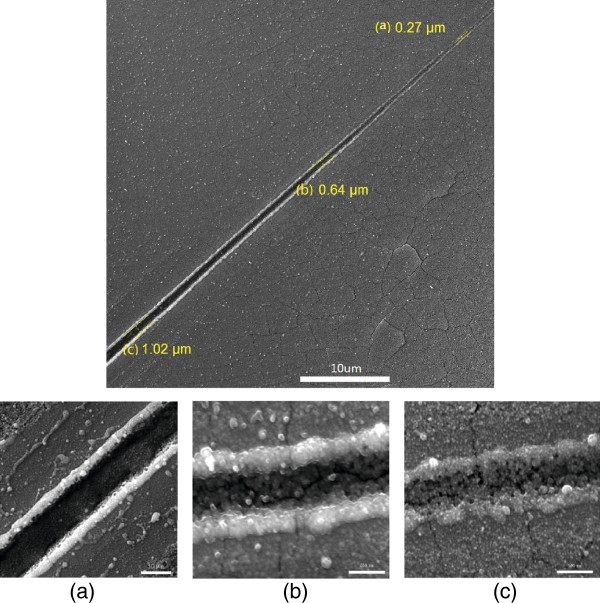
SEM image of a laser-etched TiAlSiN film and (a), (b), (c) are magnified parts.

Figure 2 shows the SEM image of the etched fiber after being dipped into the HF acid solution (47%) for about 50 minuts. Its diameter is at 5.3 μm. To study the effect of the etching process, TiAlSiN films after the laser etching are observed by SEM for surface patterning at the laser fluence of 445 mJ/cm^2^, as shown in Figure 3. After the laser treatment, the fiber has ejected from sample surfaces by short-pulse laser irradiation due to the fast thermal expansion of the fiber and/or solid surfaces. There is a groove formed on the sample at the original position of the fiber only by one laser pulse. The resultant groove is inhomogeneous in size on the laser treated surface. Its widths are 0.27, 0.64, and 1.02 μm. Some TiAlSiAl particles, which examined by Energy Dispersive Spectrometer (EDS, FEI Quanta 200 FEG, Hillsboro, Oregon, USA), bombast out from the grooves in the laser process. The changes of groove widths are because of the nonuniform fiber and the increased distance between the fiber and the surface. At the fiber end, the distance is about 0.5 μm, and the diameter reduces from 5.3 to 2.7 μm, as shown in Figure 2. These are helpful to decrease the groove width, as disscussed in the simulation section. The same laser irradiation was also induced on the clean TiAlSiN surfaces (without the fiber), but no groove was observed. This indicates that the laser etching with micro-optical fiber-enhanced irradiation can create a groove on the sample at micrometer scale. It is attributed to laser light intensity enhancement near the contact area between the fiber and sample during the irradiation.

This etching technique of the microfiber-enhanced irradiation is also attemped to process grooves on Si. A inhomogeneous groove with a minimum size of around 300 nm is also observed on the laser treated surface. As the fiber changes in the diameters, the groove dimension is relatively altered. However, the further investment needs to find out the fabrication technique and etching mechanism.

**Figure 4:**
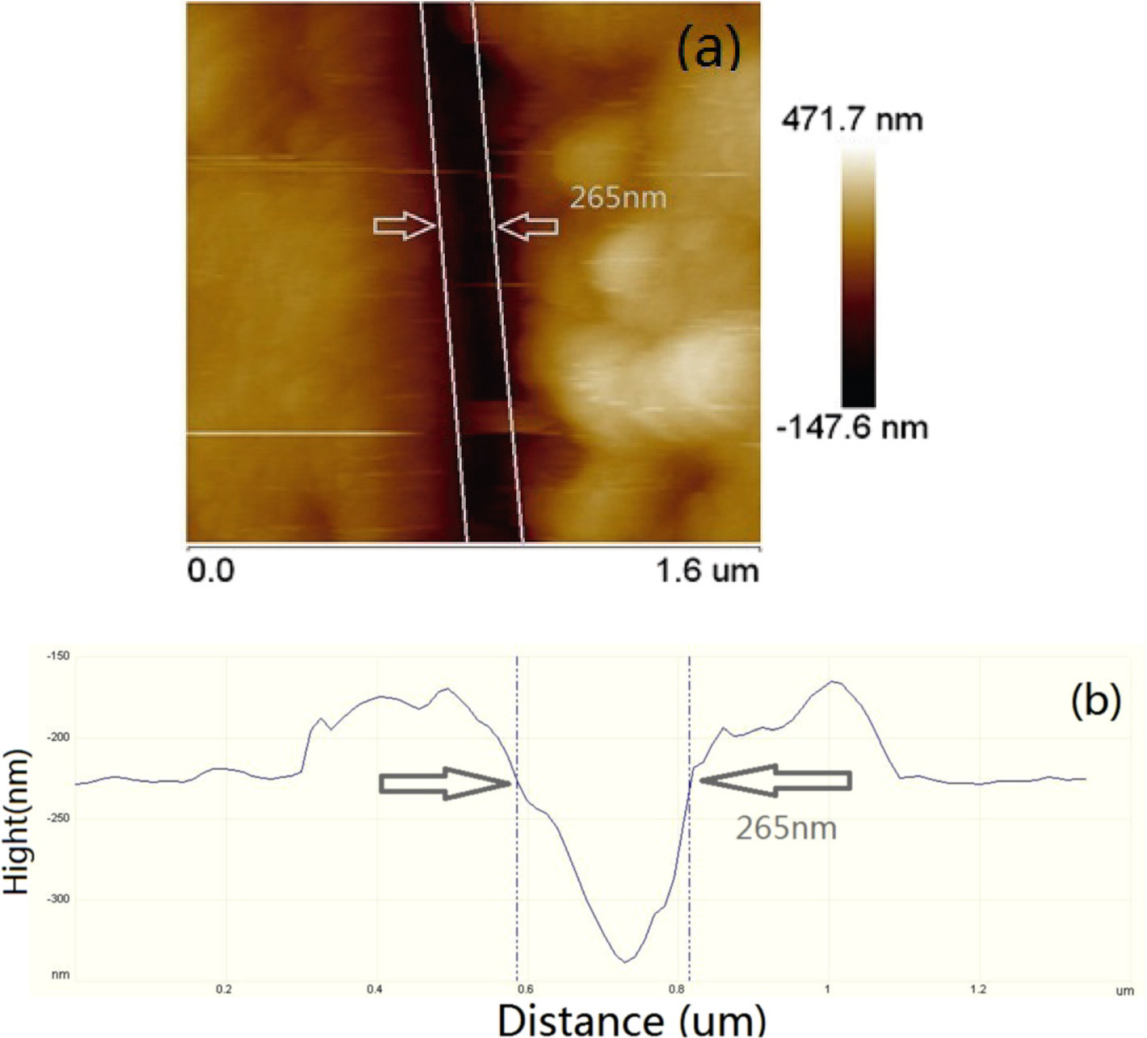
**The AFM picture of a groove and its cross section line.** AFM image (a) and its cross section line (b) of a groove formed on TiAlSiN furface, (a)for 265 nm groove; (b) is the cross section line.

Figure 4 presents an AFM picture of a groove and its cross section line. They were fabricated by using a laser at a wavelength 1.064 μm. We reveal the feature width of 265 nm (full width at half maximum, FWHM), as in Figure 4a. A cross section line of Figure 4a is shown in Figure 4b, in which the distance between two dash lines gives the FWHM value, 265 nm and the groove depth is 100 nm. This indicates that the manufacture under the diffraction limit is produced due to the light intensity enhancement. High intensity to form the microgroove is achieved because of the fiber size. This mechanism can be explained as the enhancement of light intensity near the contact area [11-13].

**Figure 5:**
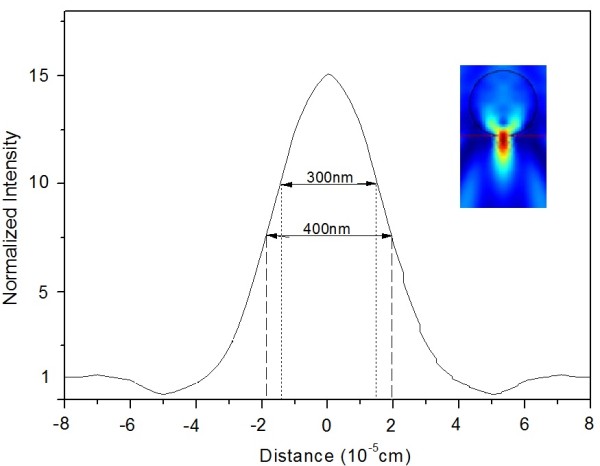
**The light intensity distributions on the substrate surface under the microfiber.** Light intensity distributions on the substrate surface under the microfiber (calculations with Maxwell equation), which is the distribution of the energy intensity perpendicular to the fiber axis. The upper right picture shows a cross section image of light intensity distribution inside and outside a 5 μm fiber illuminated by 1.06 μm laser.

Calculations of the light intensity under the fiber are carried out by solving the electromagnetic boundary problem through ’Maxwell equations’. Here, fiber models in Comsol 3.5 permit us to estimate the groove characters. Figure 5 gives the intensity distribution following the fiber's back plane and perpendicular to the wave vector of the incident wave. Y-axis presents the light intensity, as the normalized value of the z-component of the time-averaged Poynting vector. The maximum intensity is around 15× greater than the incident one. The higher intensity according to calculations was clearly observed in our experiment. Figure 5 also gives the FWHM of 400 nm in the intensity distribution under the fiber. If the light intensity between the fiber and the substrate is actually controlled over the damage threshold of TiAlSiN surface, the sub-wavelength groove can be produced by the microfiber-enhanced irradiation. Kane explained this similar result, in which the damage threshold of glass substrates can be reduced by smaller surface particle coverage [14]. Three hundred nanometer of the intensity distribution in Figure 5 is at around 10× to the incident light, which agrees with the experiment result 265 nm. It suggests that the higher resolution of the groove etching is produced by the lower incident light intensity in the microfiber system.

Two mechanisms of Rayleigh scattering and Mie scattering are proposed to explain the enhancement by the laser irradiation of particles [6]. The intensity distribution changes dramatically as the particle sizes and the interaction distance decreased. Laser procession through a small particle is different from a sphere lens focusing in a far field. In our work, the microfiber is considered as a kind of one-dimension optical component in microscale. Rayleigh scattering takes place when the fiber diameter is less than the wavelength of the light. It enhances the electric field at its sides along the direction of polarization of the incident light. Furthermore, when the diameter is equal to or greater than the incident wavelength, Mie scattering is used to examine this phenomenon. In contrast, the electric field is enhanced by several times towards the forward area of the sphere [15]. The intensity distribution shows that Mie scattering is the main reason for the enhancement of the fiber system. It is a potential application of the microfiber in microstructure fabrications. According to finite element analysis, choosing a suitable fiber size, wavelength, and incident light can produce the higher resolution in the etching process.

## Conclusions

A novel laser etching technique, in which a quartz fiber at a diameter of about 5 μm is placed on the TiAlSiN surface in order to induce the light intensity enhancement near the contact line, is investigated. We have demonstrated that the laser etching with microfiber-enhanced irradiation can be used to fabricate a groove. Its minimum width is about 265 nm. This technique is found to have advantages of simple setup and low cost. Meanwhile, the experimental results are consistent with computational simulations.

## Competing interests

The authors declare that they have no competing interests.

## Authors’ contributions

DL carried out the design and fabrication of the experimental setups and drafted the manuscript. JL participated in the fabrication of the grooves. WH established the theoretical formalism and participated in the numerical calculations. TS supervised the whole study. All authors read and approved the final manuscript.

## Authors’ information

DL is a lecturer in State Key Laboratory of Tribology, Tsinghua University. JL is a PHD student. HW is a research assistant. TS is a professor of State Key Laboratory of Tribology, Tsinghua University.
